# Comparative Study of Relevant Methods for MRI/X Brain Image Registration

**DOI:** 10.1007/978-3-030-51517-1_30

**Published:** 2020-05-31

**Authors:** Marwa Abderrahim, Abir Baâzaoui, Walid Barhoumi

**Affiliations:** 8 Digital Research Centre of Sfax, Sfax, Tunisia; 9 Institut Mines-Télécom, CNRS, Paris, France; 10 Université de Sherbrooke, Sherbrooke, QC Canada; 11 Digital Research Centre of Sfax, Sfax, Tunisia; 12 University of Sfax, Sfax, Tunisia; 13 Institut Supérieur d’Informatique d’El Manar, LR16ES06 Laboratoire de recherche en Informatique, Modélisation et Traitement de l’Information et de la Connaissance (LIMTIC), Université de Tunis El Manar, 2080 Ariana, Tunisia; 14 Ecole Nationale d’Ingénieurs de Carthage, Université de Carthage, Tunis-Carthage, Tunisia

**Keywords:** MRI/X brain image registration, Hybrid method, Standard registration tools, Brain diagnosis

## Abstract

Several methods of brain image registration have been proposed in order to overcome the requirement of clinicians. In this paper, we assess the performance of a hybrid method for brain image registration against the most used standard registration tools. Most traditional registration tools use different methods for mono- and multi-modal registration, whereas the hybrid registration method is providing both mono and multi-modal brain registration of PET, MRI and CT images. To determine the appropriate registration method, we used two challenging brain image datasets as well as two evaluation metrics. Results show that the hybrid method outperforms all other standard registration tools and has achieved promising accuracy for MRI/X brain image registration.

## Introduction

**Fig. 1.**
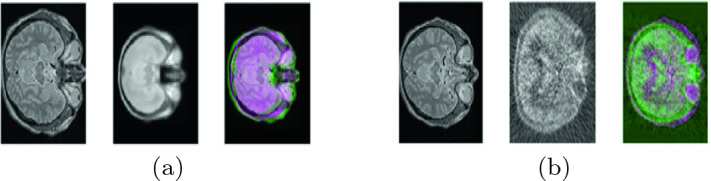
MRI/X brain image registration: (a) mono-modal MRI/MRI atlas (from left to right: MRI image, MRI atlas and superposed images), (b) multi-modal MRI/PET (from left to right: MRI image, PET image and superposed images).

Hundreds of millions of people worldwide suffer from neurological disorders, and early detection coupled with appropriate treatment can generally cure these diseases. In this context, Computer Aided Diagnosis (CAD) explains the need to design automatic and semi-automatic tools to effectively process brain medical imaging. This could help clinicians to detect affected organs in order to specify appropriate treatments. However, there are still many challenges (*e.g.* noise, resolution, partial volume effect $\ldots$) that need to be investigated. There are several brain medical imaging modalities, and each of them has a different aspect of anatomy and/or functionality. Anatomical medical imaging (*e.g.* Magnetic Resonance Imaging (MRI), Computed Tomography (CT) $\ldots$) provides information on the structure, the shape, the edge, and the contents of organs. Functional medical imaging (*e.g.* Positron Emission Tomography (PET) $\ldots$) focuses on the function of organs, tissues or cells. In clinical routines, experts generally refer to both functional and structural aspects conjointly. In particular, MRI is frequently coupled with CT, MRI atlas and PET. However, a registration step is required in order to ensure effectively the complementarity of structural and functional images. Research on registration process is driven either by the type of attributes (geometric *vs.* iconic methods), the type of transformation (rigid *vs.* non-rigid) or the involved images (monomodal *vs.* multimodal) (Fig. 1). The principle of geometric registration methods consists in extracting geometric primitives from the two images to be registered (*e.g.* points, curves, surfaces $\ldots$), whereas iconic registration methods operate directly on the intensities. Furthermore, the rigid registration methods aim to correct the geometric transformations, including translation, rotation, shear, and scaling, whereas the non-rigid registration methods are carried out using localized stretch of the images. In this type of transformation, all kinds of deformation fields can be used (*e.g.* splines, B-spline, elastic model $\ldots$) [1]. For the monomodal registration methods, the two images are coming from the same modality (*e.g.* MRI scans, CT scans $\ldots$), whilst in the multimodal registration ones, the two images come from two different modalities (*e.g.* MRI and PET, MRI and CT $\ldots$) [2]. Generally, one of the key challenges in brain image registration is its veracity. This is because of the limitations in the registration methods, which are dependent on the quality of MRI/X parameters as well as the inaccuracy on the non-linear transformations. In addition to the registration errors, several registrations methods suffer from the extensive computational cost. To circumvent these limits, atlas-based registration coupled with standard softwares (such as SPM, ITK $\dots$) are commonly used. Indeed, various studies are using this framework in order to investigate Parkinson disease [3], brain tumors using CT/MRI [4] or PET/MRI [5], and Alzheimer disease [6]. Additionally, challenges can arise where mono- and multi-modal registration is required sequentially. To this end, we evaluate a hybrid method that may handle mono- and multi-modal registration according to the same technique. In fact, this paper is dedicated to determine the best tool for mono- and multi-modal registration for MRI/X brain images, such that X refers in our case to PET, CT and MRI atlas. We compare three widely brain image registration tools (SPM, ITK-Snap, 3D Slicer) against an accurate hybrid registration method from the state-of-the-art.

The rest of this paper is organized as follows. Section 2 shows the studied registration methods. Then, we present the clinical datasets and the evaluation protocol in Sect. 3. We detail experimental results in Sect. 4. Finally, a conclusion with some directions for future work are discussed in Sect. 5.

## Registration Methods

**ITK-Snap.** Insight Segmentation and Registration Toolkit (ITK-Snap) is a popular tool for segmenting and registering medical images such as MRI, PET and CT [7]. It is an open source software widely used by clinicians and non-computer researchers. ITK-Snap allows manual and automatic medical image registration. This software groups several methods of registration based on the intensity. For the automatic registration, the similarity measures included in ITK-Snap are mutual information, cross-correlation, and intensity difference. The transformation model included is affine and rigid transformation. This tool helps the users to locally find optimal rigid and affine transformations dynamically. For the manual registration, it is enough to determine the values of *x*, *y*, and *z* for the translation, rotation, and scaling. In our case, we used the same settings as [8].

**SPM.** Statistical Parametric Mapping (SPM) is an open source software for analysing functional brain imaging data (*e.g.* fMRI, PET, SPECT $\ldots$). It uses several setting options, which are referred to the Powell optimization algorithm. These options are: objective function, separation, tolerance and histogram smoothing. For the objective function, SPM uses either mutual information, normalized mutual information, or entropy correlation coefficient for multimodal registration, and normalised cross-correlation for monomodal registration. Separation, which is the average distance between sampled points, is of 8 mm for fMRI and 12 mm for PET [9]. SPM applies Gaussian smoothing to the $256\times 256$ joint histogram. For similarity measurement, SPM includes the Nearest Neighbor, trilinear, and B-spline interpolation, and trilinear interpolation proved to be the most adequate for MRI and PET. For monomodal registration, SPM presents other parameters for estimating deformations (*e.g.* bias regularisation). Also, a mutual information-based affine registration with the tissue probability maps is used to obtain approximate alignment, with a smoothness value of 0 mm.

**Fig. 2.**
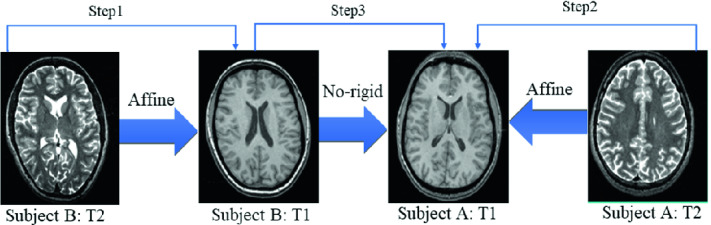
Flowchart of 3D Slicer.

**3D Slicer.** 3D Slicer [10] supports rigid, affine and deformable registration. It includes point-surface and intensity-based registration. In fact, individual intensity-based registration modules depend on the used similarity metric (mutual information and cross-correlation) and flexibility of the transformation settings (rigid, affine, B-spline and dense deformation fields) [11]. The choice of algorithms depends on the organs’ anatomy (*e.g.* brain, lungs $\ldots$), modality (multimodal *vs.* monomodal), performance (robustness *vs.* speed), and level of interaction. Besides, 3D Slicer uses parametric maps in order to align anatomical volumes. The registration process consists of three steps (Fig. 2). Firstly, it allows to align subject B: T2 according to the MRI mode T1 of the same subject. Secondly, it aligns subject A: T2 according to A: T1. Lastly, the registration is performed between the registered subject B: T1 and the fixed subject A: T1.

**Fig. 3.**
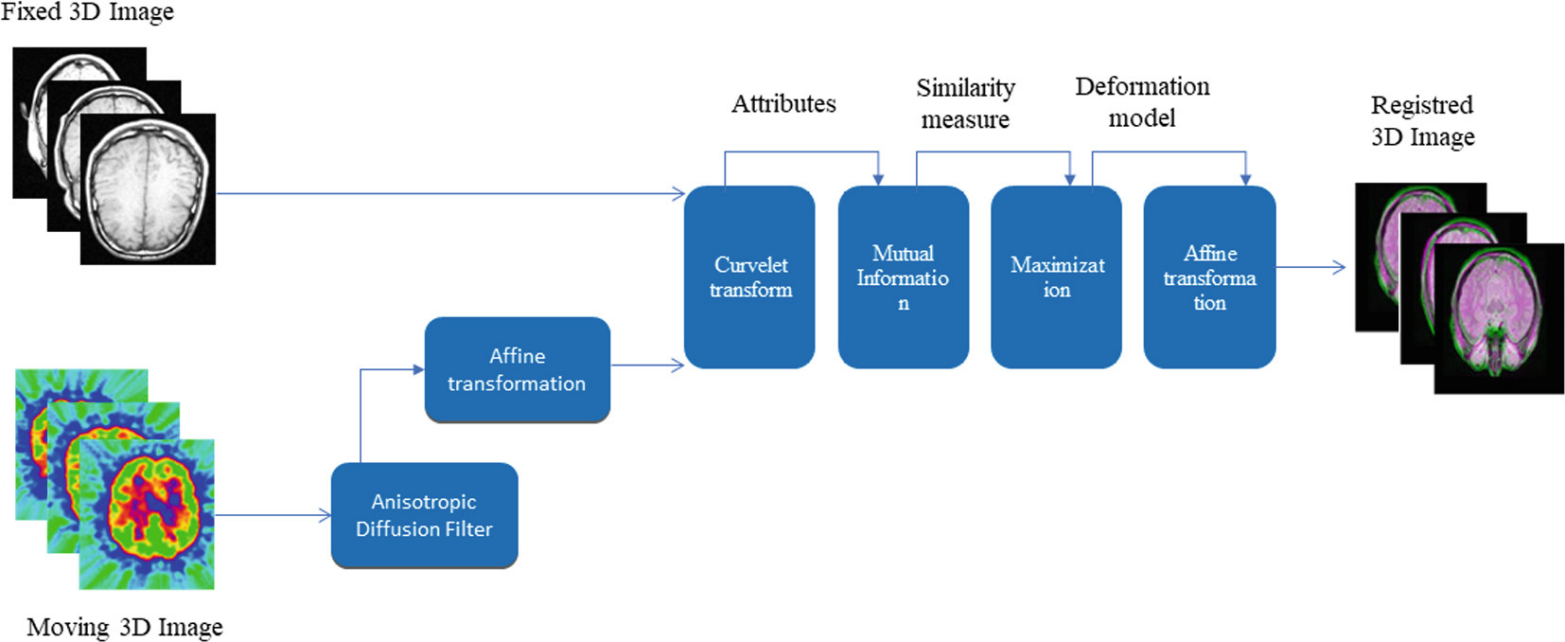
Flowchart of the hybrid method.

**Hybrid Method.** The hybrid method is a unified tool for mono- and multi-modal 3D brain image registration. In fact, we extended the multi-modal 2D brain image registration work of [2]. The method is composed of five steps (Fig. 3) and its main contribution lies in adopting adaptive mutual information based on curvelet coefficients. Firstly, an anisotropic diffusion filter [12] denoises the moving image. Secondly, an affine transformation is applied on the moving image using transformation matrices (translation, rotation, scaling and shear). Thirdly, features from the two images are extracted using curvelet transform [13], and the Gaussian probability density function [14, 15] is used to model the distribution of curvelet coefficients. Then, an adaptive mutual information, based on a conditional entropy between the coefficients of curvelet, aligns the images, and mutual information parameters are optimized using the maximum likelihood [16]. Finally, to align the moving image on the reference one, an affine transformation is adapted in order to deal with common distortions.

## Materials

In this section, we present the used 3D medical image datasets and the evaluation protocol that we adopted in order to evaluate the compared registration methods.

**Clinical Datasets.** To compare the performance of the studied methods, two datasets were investigated. The first dataset, from the Retrospective Image Registration Evaluation (RIRE) project [17], consists of eight 3D triplets of PET, MRI and CT images of brain. The MRI voxel size is of 1.25, 1.28 and 4 mm in the *x*, * y* and *z* directions, respectively. The PET voxel size is (2.59 mm, 8 mm, 8 mm) in *(x, y, z)*. MR images have been obtained using a Siemens SP 1.5 T scanner, and the PET ones with a Siemens/CTI ECAT 933/0816 scanner. The CT voxel size is equal to (0.65 mm, 0.65 mm, 4.0 mm) in *(x, y, z)*. CT images have been acquired using a Siemens Somatom Plus scanner. The second dataset is provided by the Center for Addiction and Mental Health of Canada (CAMH). It includes a collection of nine 3D images. For fixed MRI images, voxel dimensions along the *x*, *y*, and *z* axes are 0.86, 0.86, and 3 mm, respectively. These images are captured by a Signa 1.5-T scanner from General Electric Medical System. PET images are captured by a Scanditronix PET scanning system, GE 2048-15B, with *x*, *y* and *z* voxel dimensions equal to 2 mm, 2 mm and 6.5 mm, respectively.

**Evaluation Metrics.** To quantify the accuracy of the studied methods, we measured Normalized Cross-Correlation Coefficient (NCCC) (1) and Normalized Mutual Information (NMI) (2) scores. NCCC evaluates the degree of similarity between two medical images. In fact, cross correlation is less sensitive to linear changes in amplitude and illumination in the images to be compared. A high value of NCCC shows the high accuracy of the registration. Furthermore, NMI, which is a measure of the quality of the registration, is defined in terms of the entropy *H* of the image. It measures the proximity between the fixed source image $I_{f}$ and the moving one $I_{m}$. The more the value of normalized mutual information is, the more the accuracy of the registration process is.

1 $$\begin{aligned} \begin{array}{rl} NCCC = \frac{\sum _{X}^{x=1}\sum _{Y}^{y=1}\left( I_{m}\left( x,y \right) -\overline{I_{m}} \right) \left( I_{f}\left( x,y \right) -\overline{I_{f}} \right) }{\sqrt{\sum _{X}^{x=1}\sum _{Y}^{y=1}\left( I_{m}\left( x,y \right) -\overline{I_{m}} \right) ^{2}\left( I_{f}\left( x,y \right) -\overline{I_{f}} \right) ^{2}}}, \end{array} \end{aligned}$$

2 $$\begin{aligned} \begin{array}{rl} NMI =\frac{2\left( H\left( I_{f} \right) + H\left( I_{m} \right) \right) }{H\left( I_{f} \right) + H\left( I_{m} \right) +H\left( I_{f} \mid I_{m}\right) + H\left( I_{m}\mid I_{f} \right) }, \end{array} \end{aligned}$$

where, $H(\ )$ and $H\left( \ \mid \ \right)$ denote marginal and conditional entropies, respectively.

## Results

We compare qualitatively and quantitatively the studied hybrid method against the other aforementioned softwares for MRI/MRI, MRI/CT, and MRI/PET images.

**Fig. 4.**
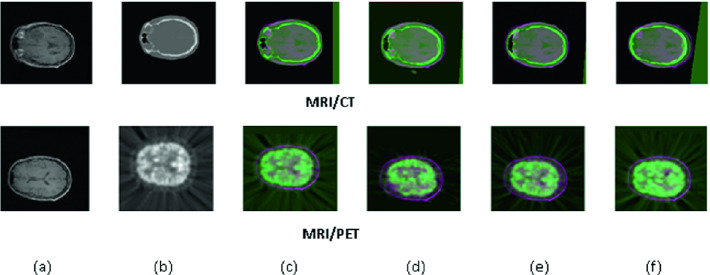
Examples of MRI/X multimodal registration: (a) MRI image, (b) X image, superposed images using (c) HM, (d) SPM (e) ITK-Snap, and (f) 3D Slicer.

**Fig. 5.**
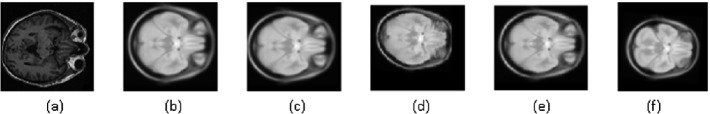
Example of MRI/MRI monomodal registration: (a) MRI image, (b) MRI atlas image, registered images using (c) HM, (d) SPM, (e) ITK-Snap, and (f) 3D Slicer.

**Qualitative Evaluation.** Figures 4 and 5 show some samples of 3D slices before and after mono- and multi-modal registrations. For the multimodal case, PET and CT refer to the moving image and the MRI image is the fixed one. Obtained results prove the performance of the Hybrid Method (HM) comparatively to SPM, ITK-Snap and 3D Slicer (Fig. 4). Monomodal registration is similar to multimodal registration, in except for the modality of the moving image (Template MRI image), which is the same of the source image (Fig. 5). We conclude that the registered images by the hybrid method show a slight improvement in the accuracy of image registration and sharpness, since that contours in these images are better represented than those of registered images using SPM, ITK-Snap and 3D Slicer. Indeed, the representative cases of the superposition of the source image and the registered one based on the hybrid method allow good boundary estimation. The visual evaluations of the outputs show that the hybrid method allows a reliable registration of MRI/PET, MRI/CT or MRI/MRI scans. This can be explained by many reasons. In fact, the use of an anisotropic diffusion filtering ensures the maximization of PET image homogeneity and the minimization of the diffusion at the edges. Furthermore, the aim behind the use of a multi-scale and multidirectional geometric transform, which is the curvelet transform, is the optimal sparse representation of smooth objects with discontinuities along curves. Then, adaptive mutual information coupled with curvelet coefficients ensures the insensitivity to the permutations of intensity while handling simultaneously the positive and negative intensity correlations.

**Fig. 6.**
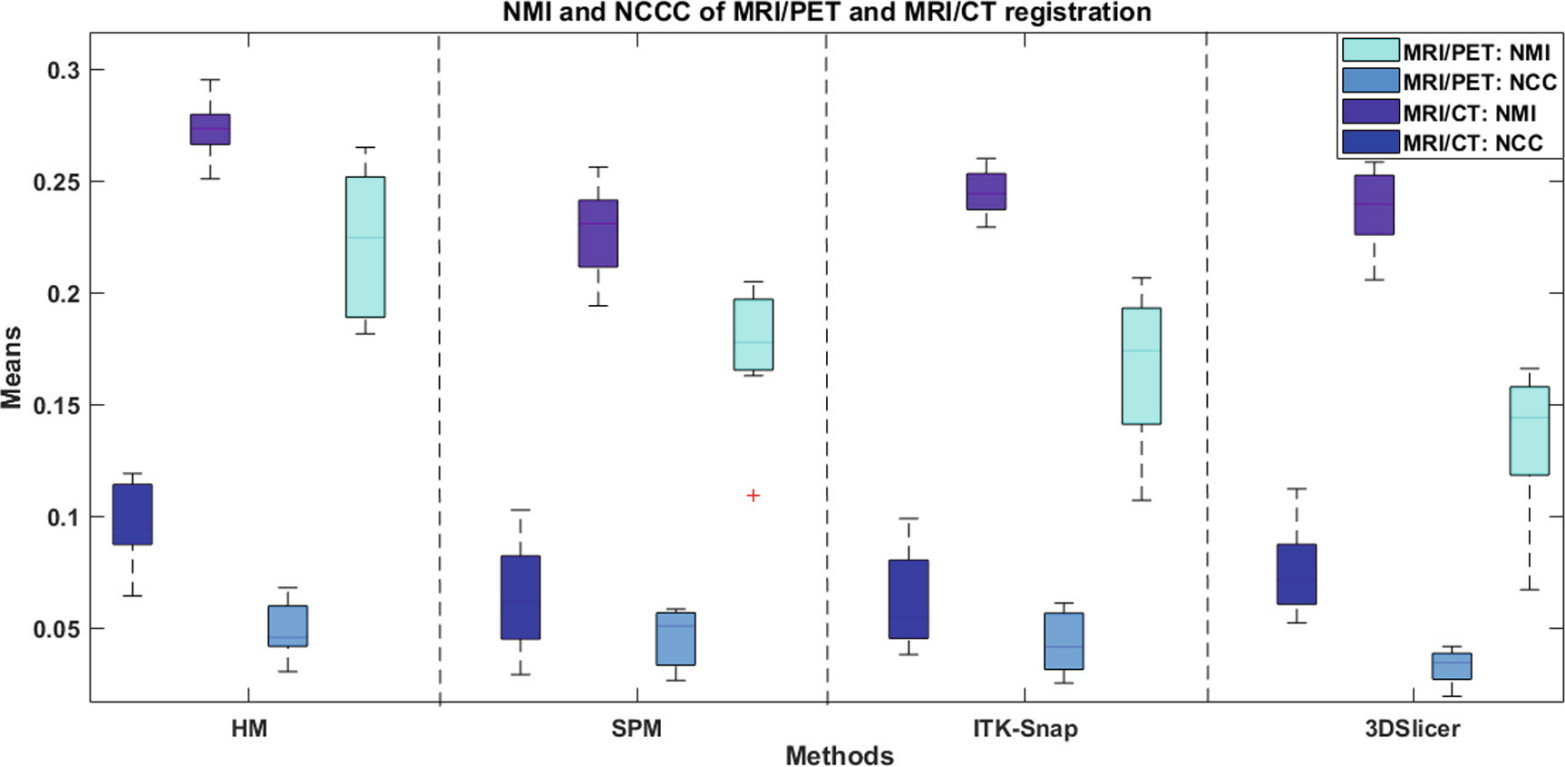
Comparing boxplot distributions of the four studied methods for the registration of MRI(PD)/PET and MRI/CT brain images from the RIRE dataset.

**Quantitative Evaluation.** The average NCCC and NMI values resulting from the analysis of different mono- and multi-modal registration methods of MRI brain images from the CAHM dataset are summarized in Table 1, whilst Table 2 illustrates MRI(T1)/PET registration results using the RIRE dataset. It is clear that both NMI and NCCC values given by the HM for the mono- and multi-modal registrations are better than those given by the other three widely used tools. The superiority of HM is confirmed by the boxplots of the four compared methods for registering MRI/CT and MRI(PD)/PET scans (Fig. 6). It should be pointed out that the nature of the images to be aligned can be very diverse and it affects considerably the choice of the registration method to be adopted. The hybrid method allows to align effectively images from the same modality as well as from different modalities. The nature of the modalities considered, as well as the type of the imaged organ, also influences the choice of the method. Likewise, the dimensionality of the input images could also be taken into consideration. Although no available registration method is perfect, research is being done to improve the results, while reducing the rate of registration error. This could increase the diagnosis confidence by improving the diagnosis accuracy.

**Table 1.**
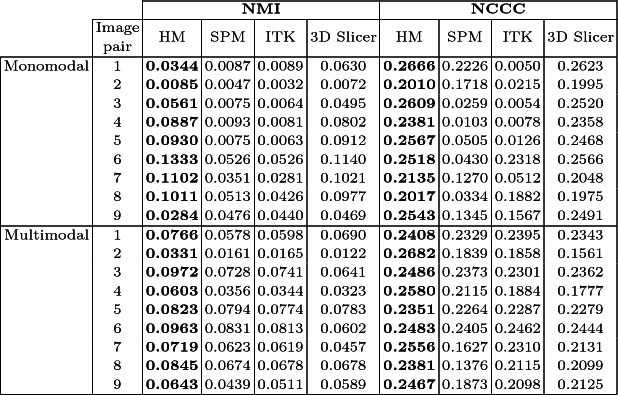
Average NMI and NCCC values resulting from the studied mono and multimodal registration methods using the CAHM dataset (best values are in bold).

**Table 2.**
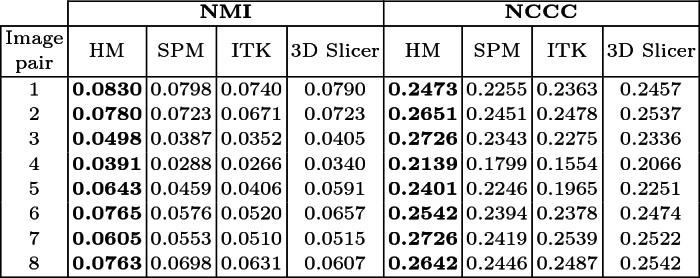
Average NMI and NCCC values resulting from the different multimodal registration methods using the RIRE dataset (best values are in bold).

## Conclusion

In this work, a comparative study of a hybrid registration method with standard registration tools is investigated for 3D brain images. The hybrid method uses mutual information based on conditional entropy for the detection of the similarity criteria, while ensuring mono- as well as multi-modal registrations. However, the standard tools use different methods to align different brain image modalities. Qualitative and quantitative evaluations show the effectiveness of the hybrid method against all other studied methods. For the brain case, rigid registration is sufficient, but for other organs, non-rigid registration is required. For that, we plan to test the hybrid method on other organs using diverse medical imaging tools while comparing it with non-rigid registration tools.
